# Why isn’t the angiotensin type 1 receptor a target in cancer?

**DOI:** 10.18632/oncotarget.15682

**Published:** 2017-02-24

**Authors:** Gavin P. Vinson

**Affiliations:** Queen Mary University of London, London, UK

**Keywords:** AT1R, breast cancer, renin-angiotensin, ACE inhibitors, losartan

The paper by Coulson et al [1] provides some of the most comprehensive evidence to date that an angiotensin type 1 receptor blocker, losartan, has highly beneficial effects on mammary tumor development and progression to invasive carcinoma in experimental animals.

**Figure 1:**
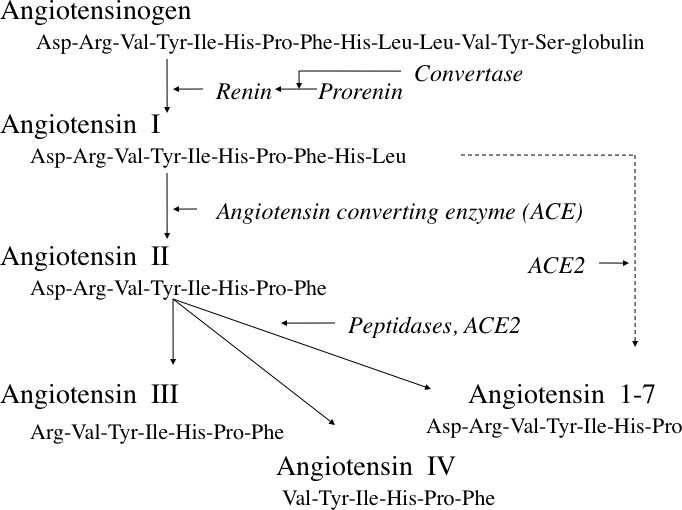
The renin-angiotensin system In normal tissue, and in cancer, the major active hormone is usually considered to be angiotensin II, though angiotensins III and IV, and angiotensin 1-7 have also been implicated (see text). Angiotensin 2-6 (not shown) may also have activity. Reproduced with permission from reference 3.

This story has taken a surprisingly long time to develop, given the known range of functions of angiotensin II and the distribution of its receptors. Angiotensin receptors are widespread in epithelia, where its stimulated actions, critical in tissue modelling as well as in functions associated with the transport of electrolytes, are well described. Accordingly, the discovery that carcinomas of many types frequently express angiotensin receptors, particularly the type 1 receptor (AT1R), should not (with hindsight) have been thought surprising. This possible link between AT1R and cancer was first suggested by their presence in breast tumors [2], and later confirmed in many tumor types. Additionally, the breast, like many other tissues, contains its own tissue-located renin/angiotensin system (RAS, see Figure 1) [3], suggesting a paracrine regulation of epithelial function that responds to localized stimuli, presumably reflecting locally-sensed demand. Overexpressed AT1R, such has been reported for some tumors, therefore potentially responds to a source of angiotensin II that is not systemically regulated.

All that does not overlook the point that there has over the years been some skepticism about the role of AT1R in cancer, and particularly about the possible therapeutic role of angiotensin converting enzyme (ACE) inhibitors (see Figure 1) or angiotensin blockers. Several studies focused on cancer incidence, and an early report that patients were protected from cancer when receiving ACE inhibitors for cardiovascular indications failed to be confirmed. Others suggested a slight increase in cancer incidence in patients receiving AT1R blockers, though this too was quickly refuted and a recent meta analysis confirmed that ACE inhibitors and AT1R blockers are substantially neutral in this regard [4]. These data of course are from patients receiving drugs for reasons other than cancer, and do not necessarily predict the outcome of any clinical trial in which they are used for cancer treatment. When combined with anti-cancer therapy, as in the studies cited by Coulson et al [1], RAS blockade has been shown to bring significant benefit.

Certainly, much evidence shows that AT1R blockers are effective in inhibiting cell growth in many cancer cell types in vitro. So how does it come about that the cancer incidence data have been equivocal? There are several possible reasons. One obvious point is that cancers rarely form a coherent group, and subpopulations may respond differently. For example, in breast cancer the subpopulation overexpressing AT1R has been reported to be no more than 20% of the total [5]. Additionally, the actions of angiotensin related peptides such as angiotensin 1-7 (see Figure 1) have been reported to be different from those of angiotensin II itself, and may oppose angiotensin II functions. Plausibly therefore ACE inhibitors could block beneficial as well as harmful actions. And again, since ACE has actions in pathways other than the RAS, ACE inhibition might also allow the accumulation of potentially harmful products such as bradykinin or substance P [6]. Another conundrum lies in the role of the angiotensin type 2 receptor (AT2R) which is also present in tumors. Although often regarded as antiproliferative, angiotensin promotes AT2R-mediated proliferation in some situations [7]. The effects of ACE inhibition on angiotensin II actions via AT2R are thus generally enigmatic.

The complex signalling that follows AT1R activation is another factor. It is certainly possible that not all of the actions of angiotensin II acting via AT1R in tumors are harmful. The actions of a monoclonal antibody to AT1R on various cell types has demonstrated that it is possible to block different signalling pathways independently, leading to the possibility of selective inhibition of harmful pathways, for example those promoting proliferation, while supporting others, such as antimetastatic pathways [8].

Finally, there remains the question of dosage. The clear in-vivo effects of AT1R blockers shown in animal cancers by various authors, and now amply confirmed by Coulson et al [1], have all been obtained using very high doses. In their paper, mice received 70mg losartan/kg/day, compared with the normal range of 20-100mg per day for adult patients. This perhaps 100-fold disparity offers another plausible explanation for the lack of effect on cancer in patients being treated for other reasons.

So, why isn’t the angiotensin type 1 receptor a target in cancer? We can conclude that certainly it should be. But perhaps not by using the drugs currently available.
